# Taxonomic study of the Oriental genus *Catullioides* Bierman, 1910 (Hemiptera, Fulgoromorpha, Tropiduchidae), with description of a new species from China

**DOI:** 10.3897/zookeys.1037.65481

**Published:** 2021-05-17

**Authors:** Hao-Yu Zhu, Fang Yu, Si-Yuan Xu, Fang-Zhou Ma, Rong-Rong Wang, Zhi-Shun Song

**Affiliations:** 1 Institute of Insect Resources and Biodiversity, School of Life Sciences, Chemistry & Chemical Engineering, Jiangsu Second Normal University, Nanjing, China Jiangsu Second Normal University Nanjing China; 2 Nanjing Institute of Environmental Sciences, Ministry of Ecology and Environment, Nanjing, China Ministry of Ecology and Environment Nanjing China; 3 Key Laboratory of Zoological Systematics and Evolution, Institute of Zoology, Chinese Academy of Sciences, Beijing, China Institute of Zoology, Chinese Academy of Sciences Beijing China

**Keywords:** Catulliini, Fulgoroidea, morphology, taxonomy

## Abstract

The tropiduchid genus *Catullioides* Bierman, 1910 is redescribed and illustrated. *Catullioides* includes two species, *C. rubrolineata* Bierman, 1910 (the type species) and *C. taishunensis* Zhu, Wang & Song, **sp. nov.** A key to the species of the genus is provided.

## Introduction

The planthopper family Tropiduchidae Stål is one of twenty-one currently recognized extant families of Fulgoroidea (Hemiptera: Fulgoromorpha) (Wang et al. 2016; Bourgoin 2021). With more than 670 species in 196 extant and extinct genera, this family is divided into two subfamilies, Elicinae Melichar and Tropiduchinae Stål (Bourgoin et al. 2019; Bourgoin 2021). Elicinae comprises four extant tribes and two extinct tribes (Gnezdilov 2013; Szwedo and Stroiński 2017; Bourgoin 2021). Distributed worldwide, Tropiduchinae comprises 17 extant tribes and two fossil tribes (Fennah 1982; Wang et al. 2016). Little research has been done on the phylogenetic relationships within Tropiduchidae (Stroiński et al. 2015), although recently, a morphological phylogeny was completed on Tropiduchini Stål (Wang et al. 2016).

In Tropiduchinae, the tribe Catulliini was first recognized by Fennah (1982) based on the type genus *Catullia* Stål and five other genera. Catulliini may be distinguished from other tribes in Tropiduchinae by the following combination of characters: frons unicarinate; pedicel of antennae with microsetae not extending to base; apical segment of rostrum very short, sometimes broader than long; hind tibiae with four lateral spines; posterior margin of mesonotum broadly rounded; forewings more than 2.5 times as long as broad; vein MP not associated with vein CuA basally; subapical cell R (C2) relatively broad at base and narrowing, usually sinuately, to apex; gonostyles symmetrical, elongate, tapering in distal half, separated from base; anterior connective lamina of gonapophyses VIII with one tooth on ventral margin, and with less than four teeth on dorsal margin; gonoplacs without teeth at apex or ventrally, longer than gonapophyses VIII [slightly modified based on Fennah (1982)]. The Catulliini taxa are mainly distributed in the Old-World tropics and subtropics, including sub-Saharan Africa, India, Sri Lanka, southern China, Japan (Ryukyu Islands), and Southeast Asia (Bourgoin 2021). Recently, the seventh genus, the fossil genus *Catulliastites* Szwedo (*nomen novum* for *Hastites* Cockerell), from the Insect Limestone (latest Eocene) of the Isle of Wight, UK, was moved to this tribe (Szwedo et al. 2019).

The Oriental genus *Catullioides* was established by Bierman (1910) for a single species, *Catullioides rubrolineata* Bierman, 1910, from Java, Indonesia. *Catullioides rubrolineata* was considered mistakenly as a synonym of *Barunoides albosignatus* (Distant, 1906) by Melichar (1914), creating the erroneous synonymy of *Catullioides* with *Barunoides* Distant (*nomen novum* for *Baruna* Distant, 1906) (Melichar 1914). Distant (1916) corrected this error and observed that “both Bierman’s description and figure define the costal membrane of the tegmina as possessing numerous transverse veins, whereas my description and figure of *Barunoides* clearly show the contrary, costal membrane without transverse veins” (Distant 1916: 53). This taxonomic error was repeated in subsequent literature recording this monotypic genus (e.g., Muir 1931; Yang et al. 1989; Hayashi 1995).

While sorting and identifying planthopper material from the collection of the Insect Explorations of Taishun, Zhejiang, China, on August 21–30, 2020, we found a second and new species of *Catullioides*, *C. taishunensis* Zhu, Wang & Song, sp. nov., which is described and illustrated in this study.

### Material and methods

The specimens studied in the course of this work are deposited in the Bernice P. Bishop Museum, Honolulu, HI, U.S.A. (**BPBM**); Institute of Zoology, Chinese Academy of Sciences, Beijing, China (**IZCAS**); and Zoological Collection, Jiangsu Second Normal University, Nanjing, China (**JSSNU**).

The post-abdomens of the specimens used for dissections were cleared in 10% KOH at room temperature for ca. 6–12 hours, rinsed and examined in distilled water and then transferred to 10% glycerol and enclosed in microvials to be preserved with the specimens. Observations, measurements, and photography were conducted under a LEICA M205 C optical stereomicroscope with a Canon EOS 5D Mark IV digital camera at the Jiangsu Key Laboratory of Biofunctional Molecules, JSSNU. Some final images were compiled from multiple photographs using the Helicon Focus 6 image stacking software and improved in Adobe Photoshop CC.

The morphological terminology and measurements used in this study follow Wang et al. (2016) for most characters and Bourgoin et al. (2015) for the forewing. Features noted in the genus description are not repeated in the species description except for clarity or additional description.

## Taxonomy

### Family Tropiduchidae Stål, 1866

#### Genus Catullioides

Taxon classificationAnimaliaHemipteraTropiduchidae

Bierman, 1910

ED7339F4-F845-514B-A58D-4FB457D214F0

Catullioides Bierman, 1910: 21. Type species: Catullioides rubrolineata Bierman, 1910; by original designation and monotypy.
Catullioides Bierman: Metcalf (1954: 58); Fennah (1982: 638); Yang et al. (1989: 74).

##### Diagnosis.

*Catullioides* may be distinguished from other genera in Tropiduchidae by the following combination of characters: vertex shorter than width, anterior margin distinctly arched, lateral carinae strongly elevated, posterior margin angularly concave, median carina complete; frons and clypeus with median carina broadly and strongly convex, intermediate carinae absent; rostrum very short and robust, apical segment abruptly truncate and concave at apex; antennae with pedicel cylindrical, with no more than 20 sensory plaque organs distributed in apical half; pronotum with anterior central part distinctly produced forwards, anterior margin strongly convex, median and intermediate carinae complete and sharp; mesonotum tricarinate, lateral carinae incurving and converging anteriad; forewings narrow and long, with nodal line, costal area narrow with numerous transverse veinlets, number of apical cells between veins RA and CuA from 14 to 16; hind tibiae with four lateral spines and seven apical teeth, hind tarsomeres I with eight apical teeth; gonostyles symmetrical, elongate, outer ventral edge strongly carinate from base to apex; periandrium symmetrical, reniform and compressed; aedeagus asymmetrical, elongate, cylindrical, with four sclerotised processes; segment X of male slender and elongate, with long lateroapical angles.

##### Redescription.

Head including compound eyes slightly narrower than pronotum (Figs 3A, 7A). Vertex (Figs 3A, 7A) broad, shorter in midline than width at base; anterior margin ridged and distinctly arched anteriad, lateral carinae strongly elevated and subparallel, posterior margin carinate and angularly concave at about 100° angle, median carina distinct and complete; disc slightly depressed. Frons (Figs 3C, 7C) large and broad, convex in midline, longer than breadth, lateral margins weakly carinate, slightly converging below antennae; median carina broadly and strongly convex, intermediate carinae absent. Frontoclypeal suture (Figs 3B, C, 7B, C) distinct and straight. Clypeus (Figs 3C, 7C) about half as long as frons, median carina broadly and strongly convex. Rostrum (Figs 3C, 7C) very short and broad, reaching to middle coxae, apical segment short, as long as breadth, abruptly truncate and concave at apex. Compound eyes (Figs 3A–C, 7A–C) oval. Ocelli (Figs 3B, 7B) small, reddish, close to eye and away from base of antennae. Antennae (Figs 3A–C, 7A–C) with scape small, ring-like; pedicel cylindrical, covered with fine setulae and no more than 20 sensory plaque organs distributed in apical half.

***Pronotum*** (Figs 3A–C, 7A–C) longer than vertex in midline, distinctly shorter than mesonotum in midline; anterior central part distinctly produced forwards with anterior margin keeled and strongly convex; disc large, strongly elevated, tricarinate and delimited by intermediate carinae, median and intermediate carinae complete and sharp, median carina with a lateral pit on each side; lateral marginal areas deeply concave with a longitudinal carina on each side from eye to tegula; posterior margin subangulately concave. Mesonotum (Figs 3A, 7A) clearly tricarinate on disc, lateral carinae incurving, converging anteriad, and reaching end of median carina. Forewings (Figs 3D, F, 7D) hyperpterous, narrow and long, membranous, without granulation, with nodal line (just past midlength); costal area present, narrower than costal cell, beyond level of tip of clavus, with numerous transverse veinlets; vein ScP+R forked basad before midlength and well basad nodal line, ScP+RA separated beyond nodal line; vein MP bifurcating into MP_1+2_ and MP_3+4_ at level of nodal line; vein CuA forked before ScP+R forking; Pcu and A_1_ veins fused into a long Pcu+A1 vein at apical 1/3 in clavus; number of apical cells between veins RA and CuA from 14 to 16. Hindwings (Figs 3E, G, 7E) hyaline, ScP+R, MP and CuA bifurcating only once; ScP+R and CuA bifurcating near apical third, anterior to bifurcation of MP; veins CuP and Pcu unbranched, running close and parallel at their base; vein A_1_ bifurcating into A_1a_ and A_1b_ near middle, A_2_ unbranched; transverse veinlets *r-m* and *m-cua1* anterior to bifurcation of MP. Legs moderately long, hind tibia with four lateral spines (rarely three with the extreme basal spine absent) and seven apical teeth; hind tarsomere I with eight apical teeth and hind tarsomere II with two lateral apical teeth.

***Male genitalia*.** Pygofer (Figs 4A–D, 8A–D), in lateral view (Figs 4A, B, 8A, B), much wider ventrally than dorsally, posterior margin more or less convex medially, without process, anterior margin produced in a pair of broad and large sclerotised processes ventrolaterally, inserted in former segment; in dorsal view (Figs 4C, 8C), dorsal margin slightly excavated to accommodate segment X. Gonostyles (Figs 4A, B, D, 8A, B, 8D) symmetrical, elongate, in ventral view (Figs 4D, 8D), inner margin more or less sinuate; in lateral view (Figs 4A, B, 8A, B), narrow at base, broadest in middle, gradually convergent and tapering toward apex, acute apically; dorsal margin irregularly sinuate, with a finger-like process raised from dorsolateral margin at base, directed dorsolaterad; outer dorsal edge with a hook-like process near basal third, directed caudad and curved ventrolaterad, acute at apex, twisted; outer ventral edge strongly carinate from base to apex. Periandrium (Figs 4E–H, 8E–H) symmetrical, moderately large, in lateral view (Figs 4F, G, 8F, G), reniform, compressed, its opening declined dorsoventrally, loosely attached to aedeagus basally. Aedeagus (Figs 4E–H, 8E–H) asymmetrical, elongate, cylindrical, sclerotised basally, and inflated apically, with four various sclerotised processes; in dorsal view (Figs 4E, 8E), two right processes produced on the membranous lobe: apical process elongate, tapering laterocaudad, basal one broad, triangular, pointed dorsocephalad; dorsal process small, somewhat triangular, directed dorsocephalad; left process large and broad, knife-like, directed laterocaudad. Segment X (Figs 4A–C, 8A–C) slender and elongate, in lateral view (Figs 4A, B, 8A, B), dorsal margin straight then declined ventrocaudad; ventral margin slightly incurved, lateroapical angles truncated apically; in dorsal view (Figs 4C, 8C), slender, expanded at base, narrowed in middle, apex deeply excavated to accommodate anal style, lateroapical angles strongly produced caudad. Anal style cylindrical, relatively small, not reaching to apex.

***Female genitalia*.** Gonocoxae VIII (Figs 5B, C, E, G) with one membranous, slender, flattened endogonocoxal processes on endogonocoxal lobe. Gonapophyses VIII (Figs 5B, C, E, F) with anterior connective lamina strongly sclerotized, narrow and straight, in lateral view, tapering distad, with five minute teeth on dorsal margin, ventral margin slightly curved dorsad at apical fourth with three large blunt teeth. Gonapophyses IX (Figs 5G, H) converging apically, suddenly protruding laterad, truncate at apex. Gonoplacs (Figs 5A–C, I) fused at basal fourth, with two sclerotized lobes fully fused together and delimited by a longitudinal membranous suture: dorsal lobe elongate and tapering caudad, ventral lobe large, longer, apical part rounded, smooth. Segment X (Figs 5A, B, D, I) very small, in lateral view (Fig. 5I), triangular, broaden caudally, caudal margin reclined caudoventrad; in dorsal view (Fig. 5D), apex excavated to accommodate anal style. Anal style (Fig. 5D) relatively small, almost as long as length of caudal margin.

##### Biology.

Collecting data show that adults of both *C. rubrolineata* and *C. taishunensis* sp. nov. were collected from *Miscanthus floridulus* (Lab.) Warb. ex Schum et Laut. (common name: giant *Miscanthus*; Poaceae), the largest of the *Miscanthus* species. It has coarse foliage with a distinct central rachis on a feathery inflorescence. *Catullioides rubrolineata* exhibits phototaxis as most specimens were collected by light trapping (see also Yang et al. 1989; Hayashi 1995), while *C. taishunensis* sp. nov. was never collected in this way.

##### Diversity and distribution.

The genus contains two species widely distributed in the Oriental region.

##### Remarks.

*Catullioides* is externally similar to the genus *Catullia* Stål, but can be separated from the it by the following features: the general color of the body, especially the broad red stripes along the median carinae of the vertex, frons, clypeus, pronotum and mesonotum; the vertex with a complete median carina and angularly concave posterior margin (median carina absent and posterior margin broadly concave in *Catullia*); and the number of apical cells between veins RA and CuA of forewings from 14 to 16 (about ten in *Catullia*).

### Key to species of *Catullioides*

**Table:** 

1	Forewings almost flat, central area of basal two-thirds and apical third dark brown to black, clavus, apices of costal area, postcostal cell, and veins C1 and C2 yellowish green in males (Figs 2C, 3D); hindwings relatively broad, ratio of length to width about 1.9–2.0:1 (Fig. 3E, G)	***C. rubrolineata* Bierman, 1910**
–	Forewings tectiform, membrane distinctly incurved, mostly fuscous to black, clavus yellowish-green to dark brown in males (Figs 6C, 7D); hindwings narrow and long, ratio of length to width about 2.6:1 (Fig. 7E)	***C. taishunensis* Zhu, Wang & Song, sp. nov.**

#### Catullioides rubrolineata

Taxon classificationAnimaliaHemipteraTropiduchidae

Bierman, 1910

3885538A-007F-58B5-88D0-93FDC1E67FFD

**Figure 1.**
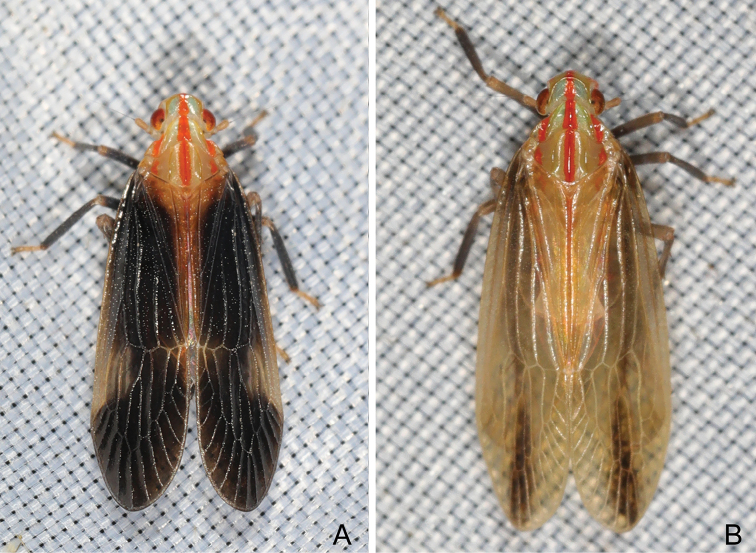
Habitus of *Catullioides rubrolineata* Bierman **A** male **B** female. Photographed by Z-S Song.

**Figure 2.**
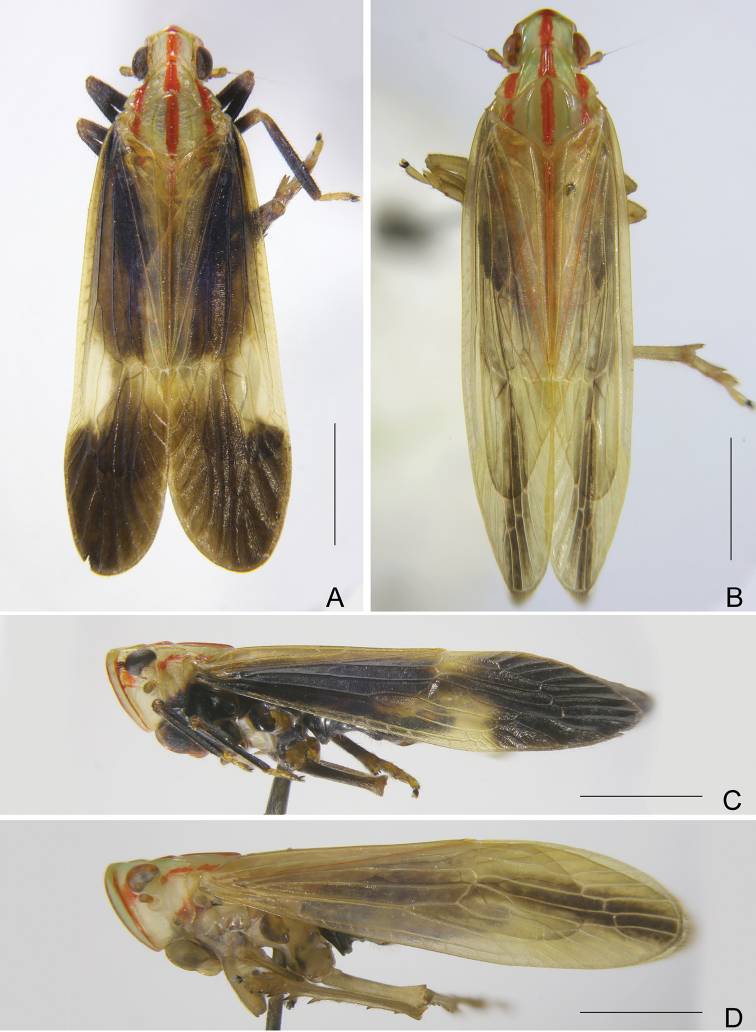
*Catullioides rubrolineata* Bierman **A** male, dorsal view **B** female, dorsal view **C** male, lateral view **D** female, lateral view. Scale bars: 2 mm.

**Figure 3.**
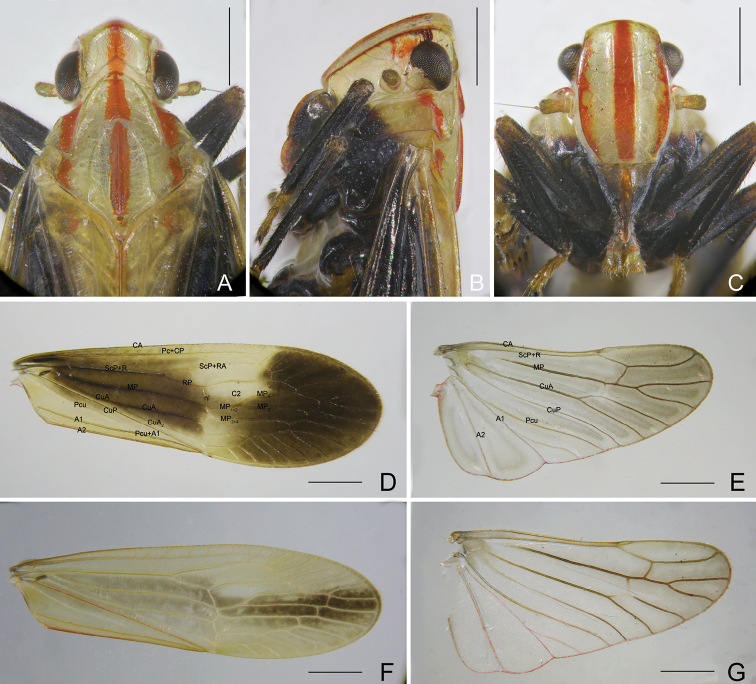
*Catullioides rubrolineata* Bierman **A** head, pronotum and mesonotum, dorsal view **B** head and pronotum, lateral view **C** head and pronotum, ventral view **D** forewing of male **E** hindwing of male **F** forewing of female **G** hindwing of female. Abbreviations: nl, nodal line. Scale bars: 1 mm.

**Figure 4.**
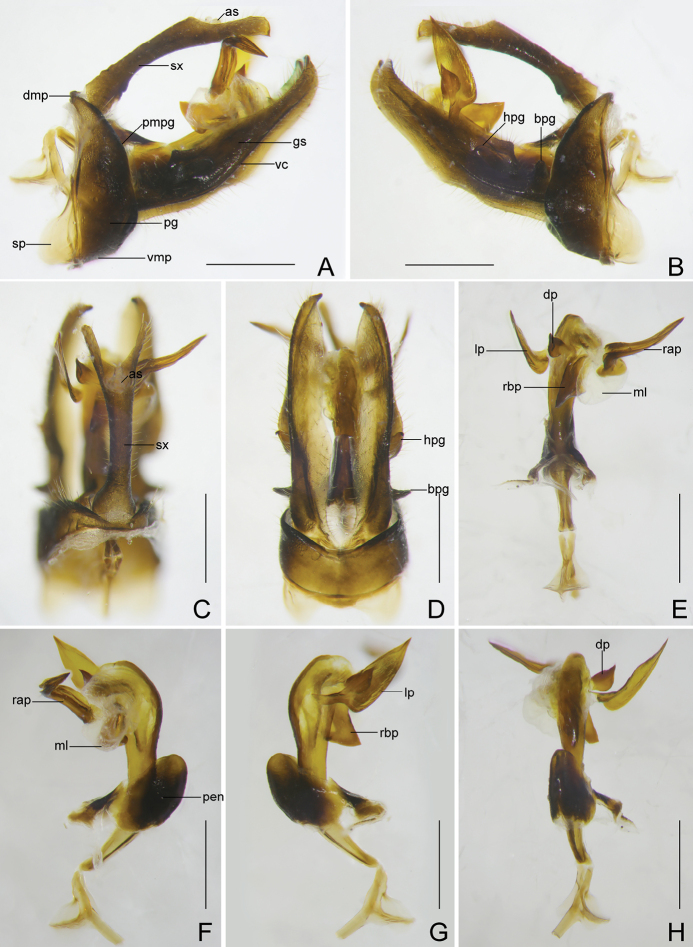
*Catullioides rubrolineata* Bierman **A** male pygofer, gonostyles, and segment X, left lateral view **B** male pygofer, gonostyles, and segment X, right lateral view **C** male segment X and pygofer, dorsal view **D** male pygofer and gonostyles, ventral view **E** aedeagus, dorsal view **F** aedeagus, left lateral view **G** aedeagus, right lateral view **H** aedeagus, ventral view. Abbreviations: as, anal style; bpg, basal process of gonostyle; dmp, dorsal margin of pygofer in profile; dp, dorsal process of phallotheca; gs, gonostyle; hpg, hook-like process of gonostyle; lp, left process of phallotheca; ml, membranous lobe of phallotheca; pen, periandrium; pg, pygofer; pmpg, posterior margin of pygofer in profile; rap, right apical process of phallotheca; rbp, right basal process of phallotheca; sp, sclerotised processes of pygofer; sx, segment X; vc, ventrolateral carina of gonostyle; vmp, ventral margin of pygofer in profile. Scale bars: 0.5 mm.

**Figure 5.**
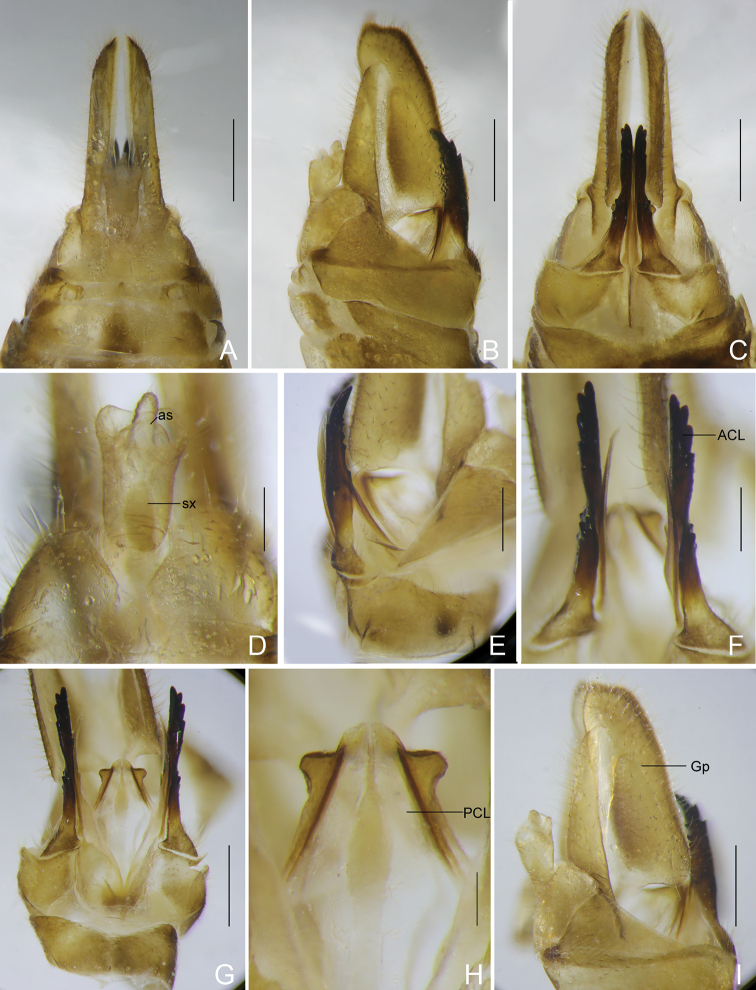
*Catullioides rubrolineata* Bierman **A** female terminalia, dorsal view **B** female terminalia, lateral view **C** female terminalia, ventral view **D** female segment X, dorsal view **E** gonapophyses VIII, lateral view **F** gonapophyses VIII, ventral view **G** gonapophyses VIII and IX, ventral view **H** gonapophyses IX, ventral view **I** gonoplacs, lateral view. Abbreviations: ACL, anterior connective lamina of gonapophysis VIII; as, anal style; Gp, gonoplacs; PCL, posterior connective lamina of gonapophysis IX; sx, segment X. Scale bars: 0.5 mm (**A–C, G, I**); 0.2 mm (**D–F, H**).

Figures 1 , 2 , 3 , 4 , 5

Catullioides rubrolineata Bierman, 1910: 22, pl. 1, fig. 9a–d.
Barunoides albosignata (Distant): Melichar (1914: 140) [error].
Catullioides albosignatus (Distant): Yang et al. (1989: 74, fig. 3); Hayashi (1995: 65, fig. 1) [error].

##### Redescription.

Body length from apex of head to tip of forewings: ♂ 8.4–9.5 mm, ♀ 9.4–10.3 mm; head length from apex of cephalic process to base of eyes: ♂ 0.7–0.8 mm, ♀ 0.8–0.9 mm; head width including eyes: ♂ 1.3–1.4 mm, ♀ 1.4–1.5 mm; forewing length: ♂ 7.0–7.7 mm, ♀ 8.1–8.6 mm.

***Coloration*.** Sexual dimorphism in general color (Fig. 1). Females distinctly paler on body than males (Fig. 2). General color pale green and red on head and thorax, and dark brown on body. Head excluding eyes, pronotum and mesonotum mostly pale green to yellowish green, broad stripes along median carinae of vertex, frons, clypeus, pronotum and mesonotum, lateral margins of frons, lateral areas of pronotum and mesonotum behind eyes red, clypeus and apical margins of paranotal lobes dark brown to black. Compound eyes red to fuscous with posterior margin pale green, ocelli purplish red. Forewings, in males (Fig. 3D), with central area of basal two-thirds and apical third dark brown to black, clavus, apices of costal area, postcostal cell, veins C1 and C2 yellowish green; in females (Fig. 3F), much paler than in males, mostly yellowish green, central area of basal two-thirds and Medial area dark brown to black. Thorax and abdomen mostly black in males (Fig. 2C); in females (Fig. 2D), much paler than in males, mostly yellowish brown.

***Structure*.** Vertex (Fig. 3A) wider than length, with ratio of length at midline to width between eyes 0.5:1. Frons with ratio of length at midline to maximum width 1.6:1 (Fig. 3C). Forewings (Fig. 3D, F) almost flat, ratio of length to width about 2.9–3.2:1. Hindwings (Fig. 3E, G) with ratio of length to width about 1.9–2.0:1.

***Male genitalia*.** Pygofer, in lateral view (Figs 4A, 4B), with posterior margin slightly sinuate, more or less convex medially, anterior margin produced in a pair of broad and large sclerotised processes ventrolaterally; in ventral view (Fig. 4D), far longer than in dorsal view (Fig. 4C), with ratio of ventral to dorsal width about 4.5:1. Gonostyles (Fig. 4A, B, D) elongate, in ventral view (Fig. 4D), inner area along ventrolateral carina less sclerotised and filmy, dorso-basal process directed dorsolaterad; in lateral view (Fig. 4A, B), ventrolateral carina strongly ridged from base to apex. Aedeagus (Fig. 4E–H) large and elongate, as long as gonostyles; in right lateral view (Fig. 4F), right apical process directed dorsad and curved laterocaudad; in left lateral view (Fig. 4G), left process large and broad, base narrow and twisted, remaining cultrate, directed laterocaudad. Segment X (Fig. 4C) slender and elongate, anal style relatively small, not reaching to apex.

***Female genitalia*** (Figs 5A–I) as in generic description.

##### Material examined.

**China**: 7♂♂, 6♀♀, Zhejiang, Taishun, Beikengdi (27°28'30"N, 119°54'28"E), 469 m, light trap, 28.viii.2020, F.Z. Ma, S.Y. Xu & H.Y. Zhu; 2♂♂, 2♀♀, same collecting locality and time, F.Z. Ma, S.Y. Xu & H.Y. Zhu (all in JSSNU); 1♀, Hainan, Shuiman, 640 m, 29.v.1960, S.F. Li; 8♂♂, 3♀♀, Fujian, Jiangle, Longqi Moutain, 200 m, 10.viii.1991, S.M. Song; 1♀, Fujian, Jiangle, Longqishan, 500 m, 13.viii.1991, X.C. Zhang; 8♂♂, 2♀♀, Yunnan, Hekou, 80 m, light trap, 7.vi.1956, K.R. Huang; 1♂, Yunnan, Xishuangbanna, Mengla, 620–650 m, 9.vi.1959, S.F. Li; 1♀, Yunnan, Jinghong, Damenglong, 30.ix.1979, J.X. Cui (all in IZCAS). **Vietnam**: 1♂, Kontum N of Pleiku, 550 m, 13.v.1960, L.W. Quate. **Laos**: 1♀, Borikhane Prov. Paksane, 20.xii.1965, native collector; 1♂, Vientiane Prov. Tha Ngone, 30.xi.1965, native collector. **Malaysia**: 1♀, Borneo, Sarawak Sadong, Kampong Tapuh, 300–450 m, 10.vii.1958, T.C. Maa (all in BPBM).

##### Host plant.

*Miscanthus floridulus*.

##### Distribution.

China (Zhejiang, Hainan, Fujian, Yunan, Taiwan); Japan (Ryukyu Islands); Vietnam; Laos; Malaysia; Indonesia.

##### Remarks.

*Catullioides rubrolineata* is newly recorded from Vietnam and Laos. Our specimens are distinctly larger than those recorded from Taiwan, China by Yang et al. (1989). Their data showed the body length of *C. rubrolineata* from Nantou, Taiwan as 5.27 ± 0.11 mm in males and 5.76 ± 0.33 mm in females (Yang et al. 1989). The type specimens of *C. rubrolineata* from Indonesia (6.5–8.0 mm) are also a little shorter than the specimens we examined (Bierman 1910). Unfortunately, we did not examine the syntypes of Bierman (1910) and the specimens of Yang et al. (1989), and identified this species based on our critical review of the literature.

#### Catullioides taishunensis

Taxon classificationAnimaliaHemipteraTropiduchidae

Zhu, Wang & Song sp. nov.

03EFD810-6696-55AB-9A28-34B5A0107932

http://zoobank.org/96FB5511-9536-49AD-A02A-E8CCA3827555

Figures 6 , 7 , 8

##### Type material.

***Holotype*** ♂, **China**: Zhejiang, Taishun, Beikengdi (27°28'30"N, 119°54'28"E), 28.viii.2020, Z.S. Song (JSSNU). ***Paratypes***, **China**: 3♂♂, same data as holotype, F.Z. Ma, S.Y. Xu & H.Y. Zhu (JSSNU); 3♂♂, same data as holotype, Z.S. Song (IZCAS).

##### Diagnosis.

The new species may be easily distinguished from *C. rubrolineata* by the distinctly incurved, non-flat forewings; the narrow and long hindwings; and the different general coloration.

##### Description.

♂, body length from apex of head to tip of forewings: 7.7–7.9 mm; head length from apex of cephalic process to base of eyes: 0.6–0.7 mm; head width including eyes: 1.2–1.3 mm; forewing length: 6.1–6.3 mm.

**Figure 6.**
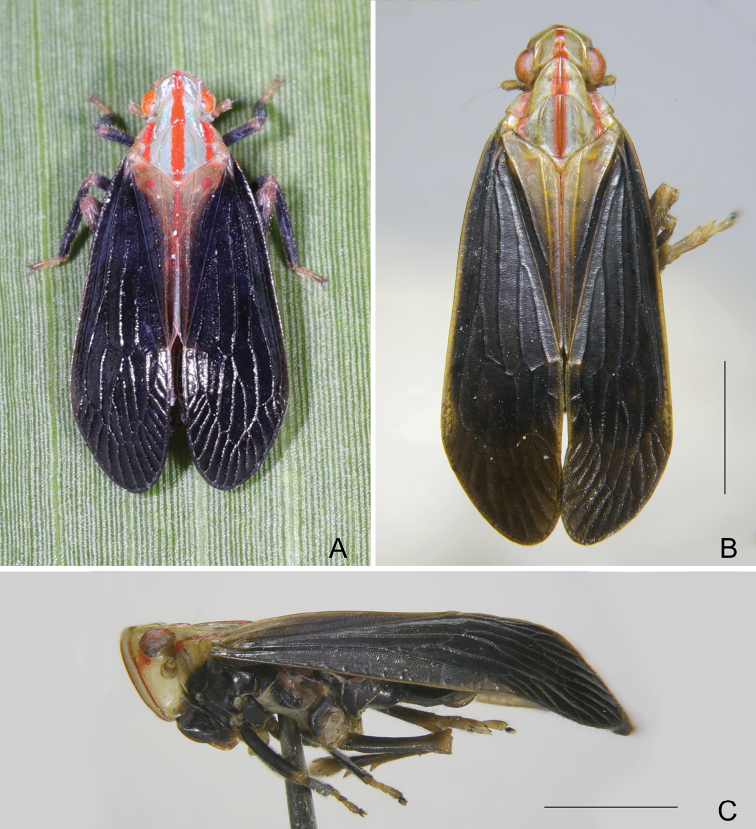
*Catullioides taishunensis* sp. nov. **A** male, photographed by Z-S Song **B** male, dorsal view **C** male, lateral view. Scale bars: 2 mm.

**Figure 7.**
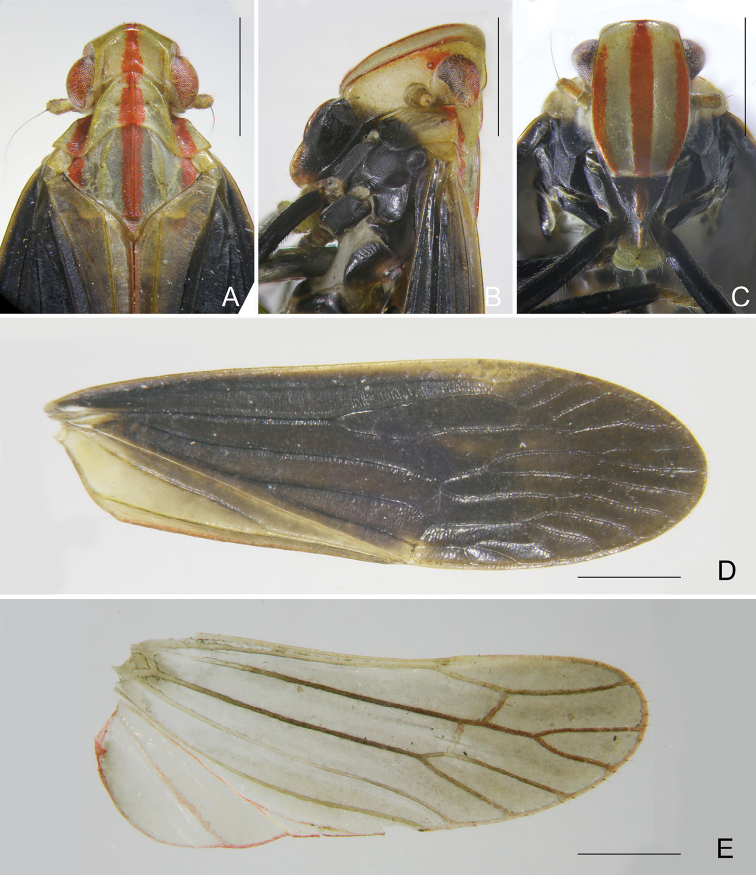
*Catullioides taishunensis* sp. nov. **A** head, pronotum and mesonotum, dorsal view **B** head and pronotum, lateral view **C** head and pronotum, ventral view **D** forewing **E** hindwing. Scale bars: 1 mm.

***Coloration*.** General color in males pale green and red on head and thorax, and black on body (Fig. 6). Head excluding eyes, pronotum and mesonotum mostly pale green to yellowish green, broad stripes along median carinae of vertex, frons, clypeus, pronotum and mesonotum, lateral margins of frons, lateral areas of pronotum and mesonotum behind eyes red, clypeus and apical margins of paranotal lobes black (Fig. 2C). Compound eyes red to fuscous with posterior margin pale green, ocelli purplish red. Forewings mostly fuscous to black, clavus yellowish green to dark brown (Fig. 7D). Lateral parts of pro- and meso-thorax black, meta-thorax yellowish green; legs black except coxae and tarsomeres yellowish green. Abdomen with terminalia mostly black.

***Structure*.** Vertex (Fig. 7A) wider than long, with ratio of length at midline to width between eyes 0.5:1. Frons with ratio of length at midline to maximum width 1.6:1 (Fig. 7C). Forewings (Fig. 7D) tectiform, membrane distinctly incurved at nodal line, ratio of length to width about 3.1:1. Hindwings (Fig. 7E) narrow and long, ratio of length to width about 2.6:1.

**Figure 8.**
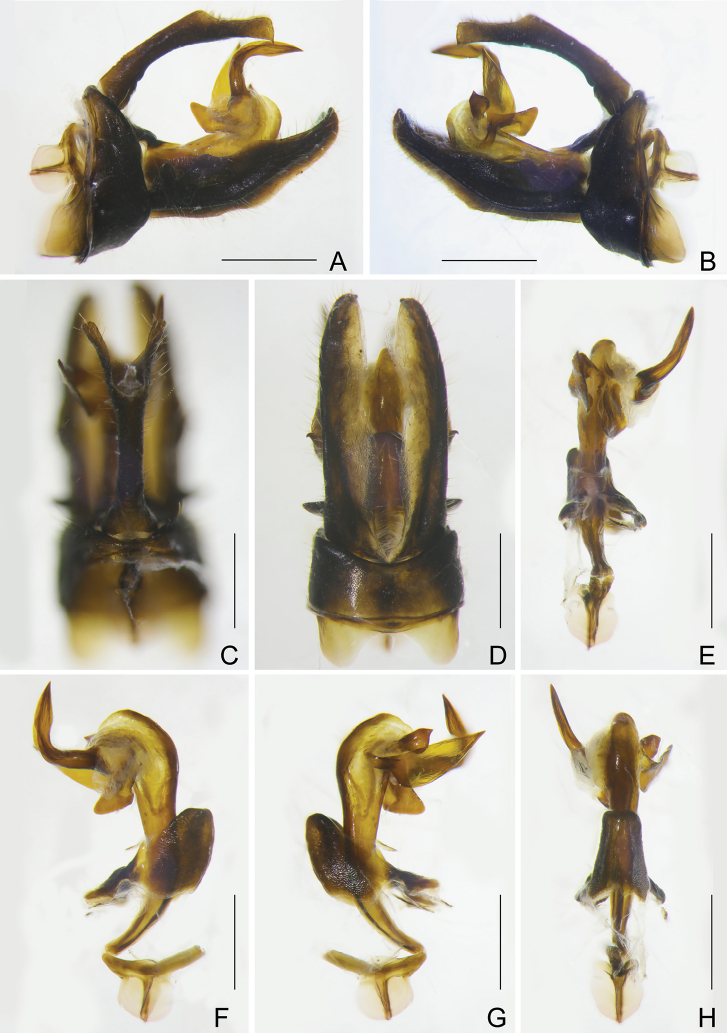
*Catullioides taishunensis* sp. nov. **A** male pygofer, gonostyles, and segment X, left lateral view **B** male pygofer, gonostyles, and segment X, right lateral view **C** male segment X and pygofer, dorsal view **D** male pygofer and gonostyles, ventral view **E** aedeagus, dorsal view **F** aedeagus, left lateral view **G** aedeagus, right lateral view **H** aedeagus, ventral view. Scale bars: 0.5 mm.

***Male genitalia*.** Similar to those of *C. rubrolineata*. Pygofer relatively narrow and small, in lateral view (Fig. 8A, B), posterior margin slightly sinuate, more or less convex medially, anterior margin produced in a pair of broad and large sclerotised processes ventrolaterally; in ventral view (Fig. 8D) far longer than in dorsal view (Fig. 8C), with ratio of ventral to dorsal width about 4.5:1. Gonostyles (Fig. 8A, B, D) elongate, in ventral view (Fig. 8D), inner area along ventrolateral carina less sclerotised and filmy, dorso-basal process directed dorsolaterad; in lateral view (Fig. 8A, B), ventrolateral carina strongly ridged from base to apex. Aedeagus (Fig. 8E–H) large and elongate, as long as gonostyles; in right lateral view (Fig. 8F), right apical process directed dorsad and curved laterocaudad; in left lateral view (Fig. 8G), left process large and broadly flat, base narrow and twisted, remaining cultrate, directed laterocaudad. Segment X (Fig. 8C) slender and elongate, anal style relatively small, not reaching to apex.

Female unknown.

##### Etymology.

The new species is named for its occurrence in Taishun, Zhejiang, China. The specific epithet *taishunensis* is to be treated as a latinized adjective in the nominative singular.

##### Host plant.

*Miscanthus floridulus*.

##### Distribution.

So far only known from Taishun, Zhejiang, China.

##### Remarks.

Bierman (1910) erected *Catullioides rubrolineata coriacea*Bierman (1910) for its smaller body and different coloration of the forewings. He did not describe and illustrate it in detail. It needs to be further studied and compared with our new species *C. taishunensis* sp. nov.

**Figure 9.**
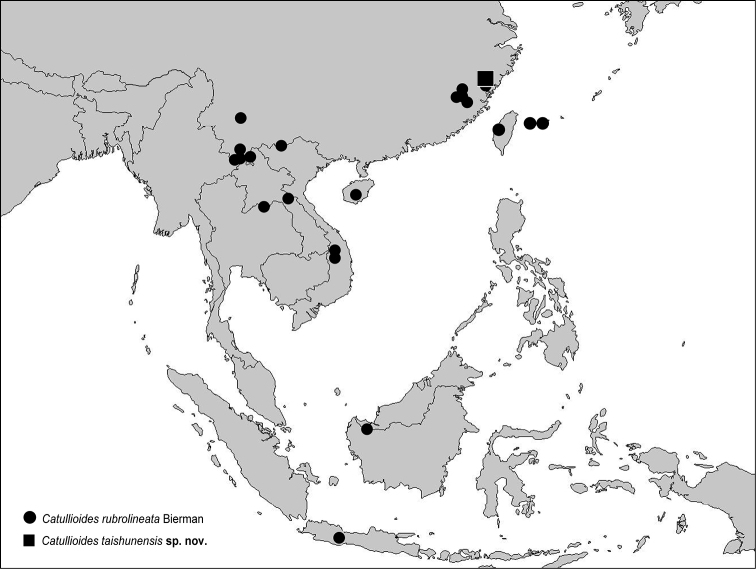
Geographical distribution of *Catullioides* species.

## Supplementary Material

XML Treatment for Catullioides

XML Treatment for Catullioides rubrolineata

XML Treatment for Catullioides taishunensis
